# DUSP1/MKP-1 represents another piece in the P2X7R intracellular signaling puzzle in cerebellar cells: our last journey with Mª Teresa along the purinergic pathways of Eden

**DOI:** 10.1007/s11302-023-09970-x

**Published:** 2023-09-30

**Authors:** Juan Carlos Gil-Redondo, María José Queipo, Yaiza Trueba, Celia Llorente-Sáez, Julia Serrano, Felipe Ortega, Rosa Gómez-Villafuertes, Raquel Pérez-Sen, Esmerilda G. Delicado

**Affiliations:** Departamento de Bioquímica y Biología Molecular, Facultad de Veterinaria, Instituto Universitario de Investigación en Neuroquímica, Instituto de Investigación Sanitaria del Hospital Clínico San Carlos, Universidad Complutense de Madrid, 28040 Madrid, Spain

**Keywords:** Astrocytes, Brain-derived neurotrophic factor, Dual-specificity protein phosphatase 1, ERK signaling, Granule neurons, P2X7 nucleotide receptor

## Abstract

The P2X7 receptor (P2X7R) stands out within the purinergic family as it has exclusive pharmacological and regulatory features, and it fulfills distinct roles depending on the type of stimulation and cellular environment. Tonic activation of P2X7R promotes cell proliferation, whereas sustained activation is associated with cell death. Yet strikingly, prolonged P2X7R activation in rat cerebellar granule neurons and astrocytes does not affect cell survival. The intracellular pathways activated by P2X7Rs involve proteins like MAPKs, ERK1/2 and p38, and interactions with growth factor receptors could explain their behavior in populations of rat cerebellar cells. In this study, we set out to characterize the intracellular mechanisms through which P2X7Rs and Trk receptors, EGFR (epidermal growth factor receptor) and BDNFR (brain-derived neurotrophic factor receptor), regulate the dual-specificity phosphatase DUSP1. In cerebellar astrocytes, the regulation of DUSP1 expression by P2X7R depends on ERK and p38 activation. EGFR stimulation can also induce DUSP1 expression, albeit less strongly than P2X7R. Conversely, EGF was virtually ineffective in regulating DUSP1 in granule neurons, a cell type in which BDNF is the main regulator of DUSP1 expression and P2X7R only induces a mild response. Indeed, the regulation of DUSP1 elicited by BDNF reflects the balance between both transcriptional and post-transcriptional mechanisms. Importantly, when the regulation of DUSP1 expression is compromised, the viability of both astrocytes and neurons is impaired, suggesting this phosphatase is essential to maintain proper cell cytoarchitecture and functioning.

## Introduction

The P2X7 nucleotide receptor (P2X7R) has been the most intensely studied nucleotide receptor and that which has sparked great debate among the community of researchers interested in the purinergic system. P2X7R, previously identified as the cytolytic P2Z receptor of immune cells, mediates a variety of activities, even including the proliferation of some cell types [1–3]. P2X7R has some peculiar features as it is a homotrimeric ion channel gated by high concentrations of ATP, almost millimolar, and it does not desensitize in the continued presence of the agonist. Moreover, it fulfills different roles depending on the type of stimulation and cellular environment. For example, tonic and submaximal activation of P2X7R promotes cell proliferation whereas sustained full activation can trigger cell death [4].

In the central nervous system, P2X7R is especially abundant in glial cells, astrocytes, oligodendrocytes, and microglia [5–7], while its presence in neurons remains unclear given the very weak expression detected in the adult brain. Neuronal P2X7R is restricted to specific locations where it may mediate neurotransmission and neuroprotection [8, 9]. However, P2X7R activity appears to be upregulated in pathological conditions [10–12], and as a result, P2X7R antagonists have generated much interest in terms of the design of drugs to treat nervous system pathologies [13, 14]. In that sense, when acting in conjunction with other purinergic signaling components, P2X7Rs modulate axonal growth and branching during neuronal differentiation [15, 16]. The participation of P2X7R in brain development has been corroborated in the P2rx7-EGFP reporter mouse model, in which the enhanced green fluorescence protein (EGFP) is expressed immediately downstream of the *P2rx7* proximal promoter [17].

P2X7R has been shown to be present in rat cerebellar cells [18, 19] and while prolonged P2X7R stimulation does not affect the survival of cerebellar astrocytes or granule neurons [20, 21], its activation does prevent the cerebellar granule neuron toxicity induced by high concentrations of glutamate [22]. The neuroprotection provoked by P2X7R stimulation was dependent on ERK1/2 activation, which induces a program of gene transcription that underlies neuronal survival. One of the genes targeted by P2X7R-activated ERK1/2 proteins is *Dusp6*, which encodes a dual-specificity protein phosphatase responsible for ERK1/2 dephosphorylation and inactivation. Indeed, DUSP6/MKP-3 is the most representative cytoplasmic ERK-specific phosphatase [23].

Dual-specificity protein phosphatases (DUSPs), also named MKPs (MAPK phosphatases), dephosphorylate the critical tyrosine and threonine residues of active MAPKs to inactivate them. Based on their sequence homology, subcellular localization, and substrate specificity, DUSPs have been classified into three groups. The first includes DUSP1, DUSP2, DUSP4, and DUSP5, which are nuclear and inducible phosphatases that exhibit broad substrate specificity. The second group consists of three cytoplasmic ERK-specific phosphatases, DUSP6, DUSP7, and DUSP9, while the third includes DUSP8, DUSP10, and DUSP16, which specifically dephosphorylate the p38 and JNK stress kinases, and that may reside in either the nucleus or cytoplasm. Although several DUSP subtypes co-exist in the same cell, they are not redundant, and they usually target substrates in different subcellular compartments [24, 25]. Indeed, DUSPs are emerging as the most potent regulators of the duration and magnitude of MAPK activity.

P2X7R and EGFR (epidermal growth factor receptor) stimulation induces ERK1/2 activation, and it regulates DUSP6 levels in a time-dependent manner in cerebellar cells. Short stimulations (0–30 min) limit the availability of protein phosphatases, and these proteins are recovered over longer periods of stimulation (1–6 h). This biphasic regulation of DUSP6 involves an early proteasomal degradation step that helps maintain ERK1/2 activity, which is followed by the synthesis of new DUSP6 protein to terminate ERK1/2 signaling [23]. As reported in other cellular systems, this biphasic regulation is triggered by its own substrates, the ERK1/2 proteins [26, 27], as activated ERK1/2 phosphorylates DUSP6 at Ser^197^ to drive its proteasomal degradation [28]. Studies performed with the allosteric DUSP6/DUSP1 inhibitor, BCI [(E)-2-benzylidene-3-(cyclohexylamino)-2,3-dihydro-1H-inden-1-one)], support these findings. Indeed, a brief exposure of cerebellar cells to this inhibitor promotes ERK phosphorylation and is correlated with a decay in DUSP6 levels [23, 29].

Following the characterization of DUSPs in cerebellar cells, we focused mainly on the nuclear inducible phosphatase DUSP1, the founder member of the DUSP family that is quite ubiquitously distributed. DUSP1 has been largely characterized in the immune system, and it is involved in the immune response and inflammation [30]. DUSP1 activity is controlled at multiple levels, transcriptional, post-transcriptional and post-translational, and as an early response gene, pro-inflammatory agents rapidly induce *Dusp1* transcription. Furthermore, DUSP1 participates in the switch between the initiation and resolution of inflammation driven by PGE_2_ in lipopolysaccharide (LPS)-activated macrophages [31]. Nevertheless, the physiological implications of DUSP1 in the nervous system are less clear, although there is evidence that it is involved in neuronal differentiation [32, 33]. The influence of BDNF (brain derived neurotrophic factor) on axonal outgrowth and branching during murine cortical neuron differentiation is dependent on *Dusp1* expression, which modulates JNK activation and cytoskeletal reorganization [34]. DUSP1 overexpression also enhances the length and branching of neurites from dopaminergic neurons, as well as preventing sustained p38 activation and the damage induced by 6-hydroxydopamine [35]. DUSP1 expression appears to be dysregulated in some neurodegenerative diseases and neurological disorders [36–39]. Considering that BDNF exerts neuroprotective effects that combat glutamate neurotoxicity in cerebellar granule neurons, DUSP1 could underlie these actions, as well as those triggered by P2X7R [22, 40]. In addition, DUSP1 may also be involved in the cytoskeletal rearrangements induced by P2X7R in cerebellar astrocytes [20, 41].

Hence, here, we studied whether P2X7R stimulation modifies DUSP1 in rat cerebellar cells, and we found that P2X7R activation induced DUSP1 expression in astrocyte and granule neurons, an effect that was more prominent in glial cells. *Dusp1* induction in astrocytes was dependent on ERK1/2 and p38 activation, whereas it only depended on ERK activation in granule neurons. EGFR activation also enhanced DUSP1 levels in cerebellar astrocytes, although this effect was weaker than that triggered by P2X7R. However, DUSP1 expression in granule neurons was under the control of BDNF. We proved that transactivation of EGFR is required for *Dusp1* transcriptional induction in cerebellar astrocytes. The data also indicate that DUSP1 levels may be modified by post-translational modifications of this protein in cerebellar cells.

## Materials and methods

### Chemicals, materials, and antibodies

Papain was purchased from Worthington (Lake Wood, NJ, USA), while the fetal calf serum (FCS), and other culture reagents were obtained from GIBCO (Life Technologies, Barcelona, Spain). The plastic Petri dishes and culture flasks were supplied by Falcon Becton Dickinson Labware (Franklin Lakes, USA), with actinomycin D, AG1478, antibiotics, Dulbecco’s modified Eagle medium (DMEM), nucleotides, epidermal growth factor (EGF), and BCI all purchased from Sigma (Madrid, Spain). BDNF was obtained from PeproTech (Bionova Científica, Madrid, Spain) and U0126 and SB202190 from Calbiochem Co. (San Diego, USA). The anti-MKP-1 and the specific antibodies against MAP kinases, phospho-ERK1/2 (Tyr^204^), were purchased from Santa Cruz Biotechnology (Santa Cruz, CA, USA), while the anti-phospho-p38 antibody was from Abcam (Cambridge, UK). The anti-phospho-Ser^323^ MKP-1 and anti-phospho-Ser^359^-MKP-1 antibodies were obtained from Signalway Antibody (Washington, DC, USA), while the antibodies against alpha and beta III-tubulin, and GFAP, as well as DAPI (4′,6-Diamidine-2′-phenylindole dihydrochloride) were from Sigma (Madrid, Spain). The anti-rabbit GFAP (glial fibrillary acidic protein) was from Dako (Denmark), while the secondary horseradish peroxidase (HRP)-conjugated anti-mouse antibodies were from Santa Cruz Biotechnology, with the anti-rabbit and anti-goat equivalents from Dako. Anti-mouse FITC and anti-rabbit Cy3 were purchased from Jackson ImmunoResearch (Suffolk, UK), the protease cocktail inhibitor was obtained from Roche (Madrid, Spain), and all other reagents were routinely supplied by Sigma/Merck (Madrid, Spain).

### Ethic statements

All the animal procedures were carried out at the Complutense University in accordance with European and Spanish regulations (2010/63/EU; RD 1201/2005; RD 53/2013) and following the guidelines of the International Council for the Laboratory Animal Science. The experimental protocols were approved by both the Committee for Animal Experimentation of UCM and that of the Regional Government of Madrid (PROEX 178.5/2020), with all the assays designed to minimize the number of animals used while maintaining statistical validity.

### Primary cerebellar cell cultures

Purified cultures of cerebellar granule neurons and astrocytes were prepared as described previously [23]. Briefly, the cerebella were removed aseptically from Wistar rat pups (P7) and digested with papain. For neuronal cultures, the dissociated cells were resuspended in neurobasal medium supplemented with B-27, 20 mM KCl, 2 mM glutamine, 100 U/mL penicillin, 0.1 mg/mL streptomycin, and 0.25 µg/mL amphotericin B, and they were plated at a density of 200,000 cells/cm^2^ in poly-d-lysine (0.1 mg/mL) pre-coated plastic Petri dishes or on glass coverslips. To avoid glial cell proliferation, 5 µM AraC (cytosine arabinoside) was added to the cultures 24 h after plating, and the cells were maintained for 7–8 days in a humidified incubator at 37 ºC in an atmosphere of 5% CO_2_.

To obtain purified astrocyte cultures, dissociated cerebellar cells were resuspended in DMEM containing 10% (v/v) FCS, 25 mM glucose, 2 mM glutamine, 100 U/mL penicillin, 100 mg/mL streptomycin, and 2.5 µg/mL amphotericin. They were plated in culture flasks at a density of 70,000 cells/cm^2^ and maintained in culture until they reached confluence (approximately 10–12 days), replacing the medium every 3–4 days. Astrocyte cultures were depleted of microglial cells by orbital shaking. Astrocytes were then detached from the culture flasks by trypsin digestion and seeded onto culture plates. For Western blotting and quantitative real time-PCR (RT-qPCR) studies, cells were plated in Petri dishes at a density of 50,000 cells/cm^2^, and for immunocytochemistry, they were plated on glass coverslips in 35-mm Petri dishes at a density of 40,000 cells/cm^2^. The culture medium was routinely replaced with DMEM containing 1% FCS 24 h after plating, and astrocytes were used the following day.

### Culture treatments and cellular lysate preparation

Cells were stimulated by adding the effectors to the culture medium, and they were maintained in the incubator for the required times. The incubations were stopped by removing the medium prior to lysing the cells with cold lysis buffer: 20 mM MOPS (pH 7.2), 50 mM NaF, 40 mM β-glycerophosphate, 1 mM sodium orthovanadate, 5 mM EDTA, 2 mM EGTA, 0.5% Triton X-100, 1 mM PMSF, and a protease inhibitor cocktail. For viability studies, the MTT tetrasodium salt [3-(4,5-dimethythiazol-2-yl)-2,5-diphenyl tetrazolium bromide] (Sigma), was added at 0.5 mg/ml directly to the culture media 24 h after the DUSP inhibitor, and maintained for 2 h at 37°C. Then, solubilization solution (10% Triton X-100 plus 0.1 N HCl in anhydrous isopropanol) was added and the samples were collected and measured spectrophotometrically at 570 nm. Values were normalized with respect to that obtained from untreated cells, considered as 100% survival.

### Western blotting

Total cell lysates (15–30 µg protein) were resolved by SDS-PAGE and transferred to PVDF membranes as described previously [23]. The membranes were blocked in TBS (Tris-buffered saline) containing low-fat milk powder (5%), and then probated overnight at 4 ºC with the primary antibodies diluted in TBS containing 0.1% Tween-20 with or without bovine serum albumin (3% BSA w/v). The primary antibodies were used at the following dilutions: anti-phospho-ERK1/2, anti-phospho-p38, anti-MKP1 and anti-phospho-Ser^323^-MKP1 (1:500), anti-phospho-Ser^359^-MKP1 (1:1,000) and anti-GAPDH, anti-alfa, or beta III tubulin (1:10,000). Antibody binding was detected for 1 h at room temperature with anti-mouse (1:2,000) or anti-rabbit (1:1,000) HRP-conjugated secondary antibodies and visualized by ECL (kit Western Lighting ECL PRO, Perkin Elmer, Madrid, Spain). Chemiluminescence images were obtained on a ImageQuant LAS 500® system and quantified by densitometry using the ImageQuantTL software.

### RT-qPCR

Total RNA was extracted from the cultured cells using the Speedtools Total RNA extraction kit (Biotools) and 1 μg of the DNAse-treated RNA (Turbo-DNA free: Ambion) was then used for cDNA synthesis, according to the manufacturer’s instructions (TaqMan Reverse Transcript Reagents, Applied Biosystems kit). Quantitative real time PCR (RT-qPCR) reactions were performed using the LuminoCt® pPCR Ready Mix™ (Sigma), 5 µl of cDNA, and the gene-specific primers (Universal ProbeLibrary Probes labeled with FAM: Roche Applied Science): TCAACGAGGCGATTGACTTT and TGGCAGTGCAAAACACC for *Dusp1* and CCCCTCTGGAAAGCTGTG and GGATGCAGGGATGTTCT for *Gapdh*. Fast thermal cycling was performed using a StepOnePlus™ Real-Time PCR System (Applied Biosystems) as follows: predenaturation at 95 °C for 20 s, followed by 40 cycles each of 95 ºC for 1 s and 60 °C for 20 s. The data were normalized to *Gapdh* expression, and the results were expressed relative to the control conditions in the absence of any stimulation.

### Immunocytochemistry

Cells plated onto coverslips were stimulated as described previously, washed with PBS, and fixed in 4% paraformaldehyde for 15 min at room temperature. After washing three times with PBS, the cells were permeabilized with 0.2% Triton X-100 and blocked with 2% BSA in PBS for 1 h at room temperature. The cells were then incubated overnight at 4ºC with the primary antibodies anti-MKP1, anti-pERK or anti-pp38 (1:100), and anti-GFAP (1:500). Subsequently, the cells were washed with PBS and incubated with FITC (1:200) or Cy3 (1:400) conjugated secondary antibodies for 1 h at room temperature. The nuclei were counterstained with DAPI (Invitrogen, Barcelona, Spain), and the coverslips were mounted onto glass slides using Aqua-Poly/Mount (Polysciences Europe GmbH, Bergstrasse, Germany). Confocal images were acquired on a TCS SPE microscope from Leica Microsystems (Wetzlar, Germany), and they were analyzed using the ImageJ and Leica LAS AF Lite software.

### Statistical analysis

The data are shown as the mean ± standard error of the mean (SEM) of a minimum of three experiments performed with cells from different cultures. Statistical differences between two groups of data were assessed using a *t* test, and a *p* value lower than 0.05 was considered significant. Multiple comparisons were performed with one-way analysis of variance, applying a Dunnett’s post-test analysis when a significant effect was evident (*p* < 0.05). All analyses were carried out with GraphPad Prism 5 (GraphPad Software Inc.).

## Results

### Short-term exposure to BCI, the DUSP1/DUSP6 inhibitor, modifies the levels of DUSP1 in cerebellar cells

**Fig. 1:**
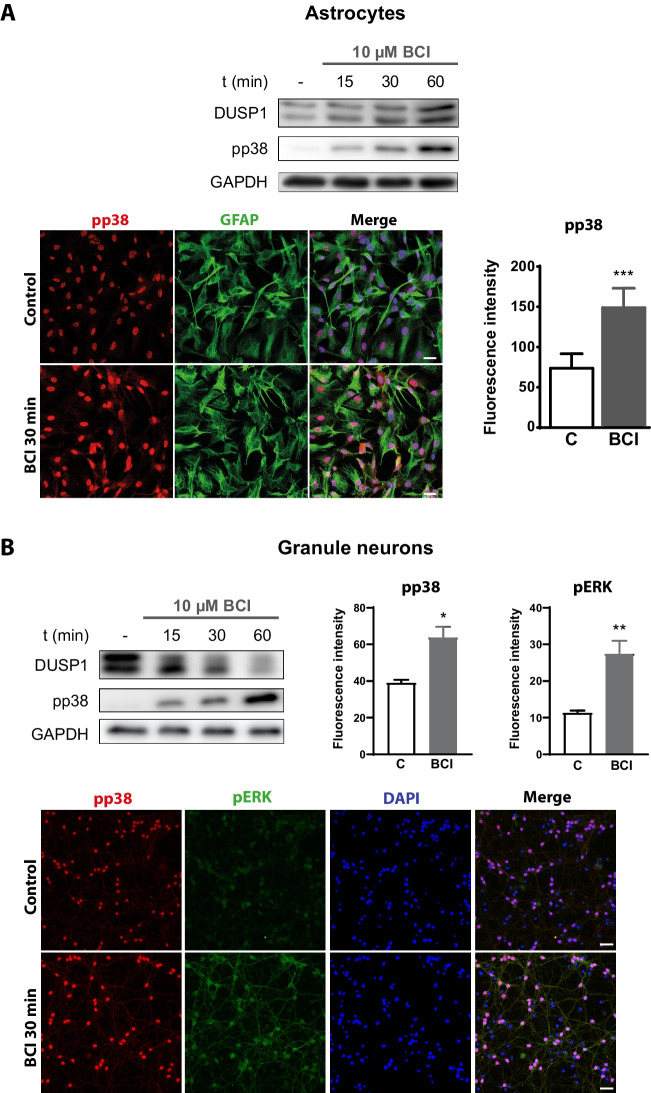
Short incubations with the DUSP6/DUSP1 inhibitor BCI regulate endogenous DUSP1 levels and p38 phosphorylation in cerebellar astrocytes and granule neurons. Cerebellar cells were incubated with BCI (10 µM) for different times (15–60 min), and the presence of the DUSP1 protein and phospho-p38 in astrocytes (**A**) or granule neurons (**B**) was assessed in immunoblots of total cell lysates. The blots shown are representative of the results obtained in three independent experiments performed on different cultures. Phospho-p38 was also detected by immunofluorescence in cells plated at low density on coverslips and maintained in the presence or absence of BCI (10 µM, 30 min), as described in the “Methods”. Representative images of astrocytes (**A**) stained for phospho-p38 and of granule neurons stained for phospho-p38 and phospho-ERK1/2 (**B**). The scale bars represent 50 µm. The graphs represent the quantification of the fluorescence intensity obtained normalized to the corresponding DAPI staining and the data are presented as the means ± SEM from four independent experiments performed on different cultures: ***p < 0.001; **p < 0.01; *p < 0.05

As indicated previously, a short exposure of cerebellar astrocytes and granule neurons to BCI elicited a time-dependent increase in ERK1/2 phosphorylation, which was inversely correlated to the decay in the basal levels of DUSP6 protein [23]. Since ERK1/2 activation might also modulate DUSP1 expression, we analyzed the effect of BCI on the DUSP1 protein. Short exposures to BCI (10 µM, 15–60 min) enhanced the DUSP1 levels in cerebellar astrocytes while having the opposite effect in granule neurons (Fig. 1). These effects could reflect differences in MAPK activation, although BCI led to an increase in p38 phosphorylation over time in both glial cells and granule neurons. Moreover, p38 phosphorylation in response to BCI was also confirmed by immunocytochemistry. There was a noticeable enhancement in nuclear phospho-p38 immunostaining in cerebellar cells treated for 30 min with BCI (10 µM), co-localizing with DAPI staining. The phospho-p38 immunostaining in granule neurons differed significantly from that observed for phospho-ERK immunolabeling in the same experimental conditions. As previously reported, phospho-ERK immunolabeling was predominant in the soma and along the fibers of cerebellar granule neurons [23], suggesting phosphatases responsible for MAPK dephosphorylation have distinct locations. Our working hypothesis was that DUSP1 could be one of the protein phosphatases responsible for MAPK inactivation in the nuclear compartment of cerebellar cells. Thus, we assessed if MAP kinases were implicated in the regulation of DUSP1 activity in the two cellular models. As P2X7R and EGFR stimulation promotes ERK1/2 activation in astrocytes and granule neurons, we examined whether such stimulation modified nuclear DUSP1 expression.

### P2X7 and EGF receptor stimulation induces DUSP1 expression in rat cerebellar astrocytes

**Fig. 2:**
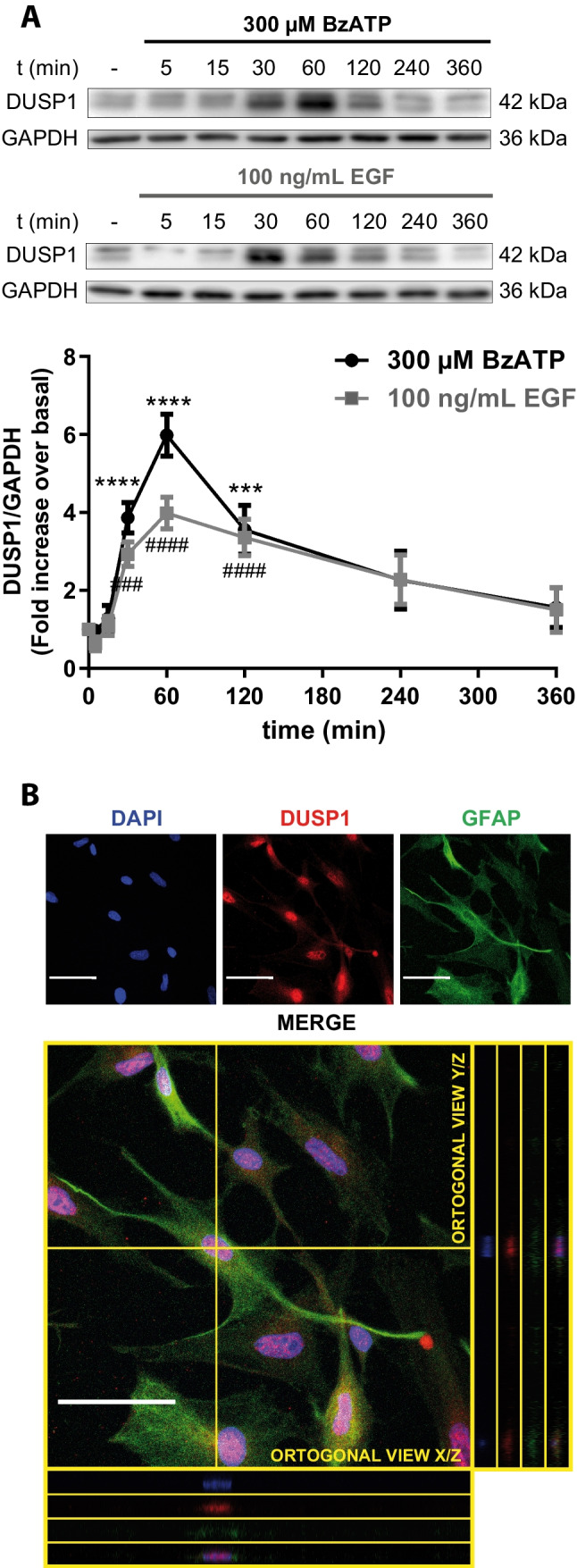
BzATP and EGF stimulation modifies the endogenous levels of DUSP1 protein in rat cerebellar astrocytes in a time-dependent manner. Cerebellar astrocytes were stimulated with BzATP (300 µM) or EGF (100 ng/mL) for the times indicated, and DUSP1 and GAPDH were assessed in western blots of the total cell lysates. **A** Immunoblots of a representative time-course experiment for each effector in which the quantification of DUSP1 is shown relative to the basal levels in control cells (unstimulated cells) and normalized to GAPDH. The data represent the means ± SEM from at least three independent experiments performed on different cultures. ****/####*p* < 0.0001; ***p < 0.001; ##*p* < 0.01. **B** Representative immunofluorescence images showing nuclear DUSP1 immunostaining. Astrocytes plated at very low density were fixed, and the DUSP1 and the astroglial marker GFAP in the cells were detected by immunofluorescence. Scale bars represent 50 µm

Cerebellar astrocytes were exposed to the BzATP or EGF for different times (0–6 h), analyzing the DUSP1 protein from these cells in Western blots. Astrocyte stimulation with BzATP (300 µM) promoted a rapid and transient increase in DUSP1 (Fig. 2A), with maximal 5–6 fold increases observed after a 1-h stimulation relative to the basal levels, which receded over longer exposures to this agonist. Similar EGF (100 ng/mL) stimulations of astrocytes also increased DUSP1 levels in an analogous pattern, with maximal phosphatase protein again achieved after a 1-h stimulation but at a lower magnitude than BzATP. DUSP1 was also detected by immunocytochemistry (Fig. 2B), with intense nuclear DUSP1 labeling co-localizing with DAPI staining. This transient expression and the nuclear localization were consistent with the regulation of an immediate early gene (IEG) and the inducible nature of the protein phosphatase.

**Fig. 3:**
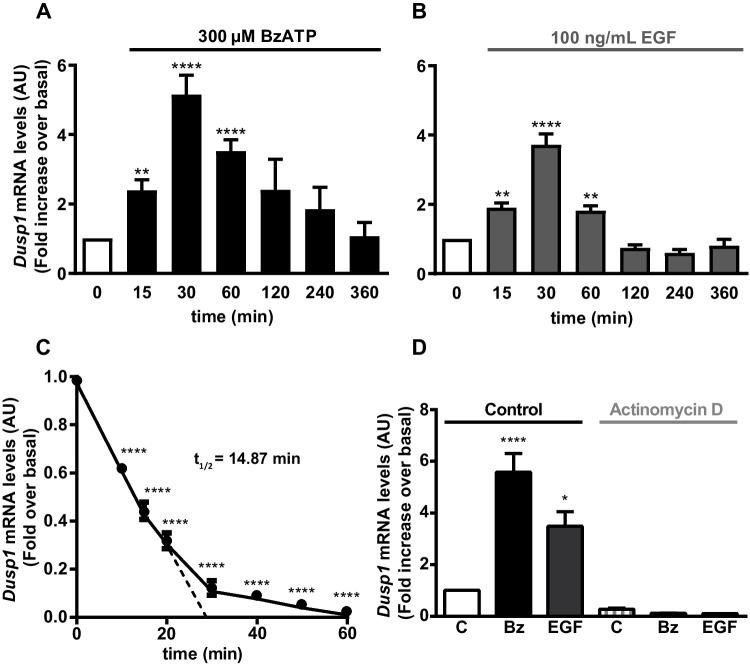
BzATP and EGF stimulation enhances *Dusp1* mRNA expression in cerebellar astrocytes. Cells were stimulated with the nucleotide (300 µM, **A**) or the growth factor (100 ng/mL, **B**) for the times indicated, and the total RNA isolated from the cells was then reverse transcribed to assess the presence of *Dusp1* and *Gapdh* transcripts by qPCR. **C** *Dusp1* expression is regulated at the transcriptional level. Astrocytes were treated with actinomycin D (10 µM) for the times indicated, and the *Dusp1 and Gapdh* mRNA expressed was quantified. The half-life (t_1/2_) of the *Dusp1* mRNA was calculated by linear regression. **D** Cells were pretreated with actinomycin D (10 µM) for 30 min before stimulating them for 1 h with BzATP (300 µM) or EGF (100 ng/mL) and quantifying the *Dusp1* and *Gapdh* transcripts as indicated previously. *Dusp1* expression was normalized to that of *Gapdh* and the data are presented as the means ± SEM from three independent experiments performed on different cultures: ****p < 0.0001; **p < 0.01; *p < 0.05

To determine whether the increased levels of this protein phosphatase were due to transcriptional induction, *Dusp1* transcripts were quantified by RT-qPCR. Exposure to BzATP and EGF rapidly increased *Dusp1* transcription, reaching a maximum after 30 min and quickly returning to basal levels (Fig. 3). *Dusp1* was more strongly induced by BzATP than by EGF. The half-life of *Dusp1* transcripts was determined by incubating cells with actinomycin D to block *de novo* gene transcription, which rapidly decreased the basal levels of *Dusp1* transcripts. These transcripts had a half-life of 14.87 min, which was not affected by any of the effectors (Fig. 3C). Moreover, pretreatment with actinomycin D completely blocked the increase in *Dusp1* mRNA transcripts promoted by BzATP and EGF (Fig. 3D).

### ERK and p38 activation are required for *Dusp1* expression in cerebellar astrocytes

**Fig. 4:**
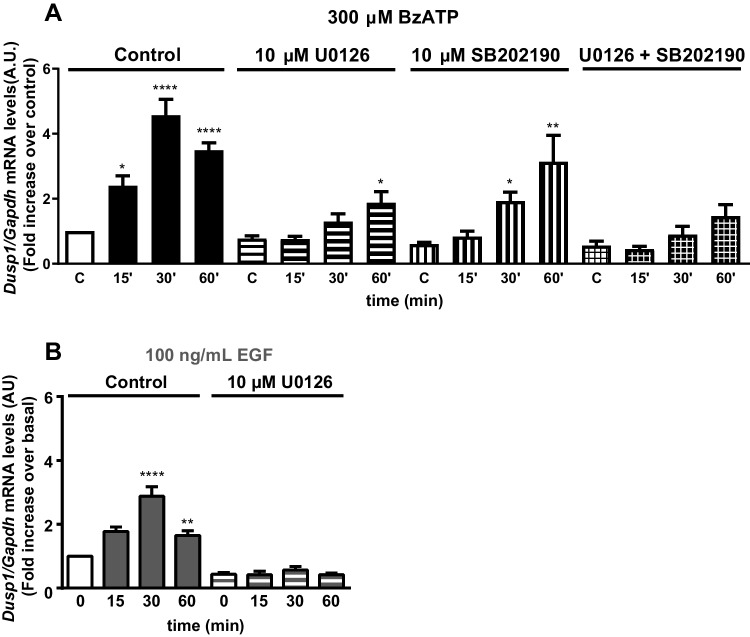
The induction of *Dusp1* transcription in cerebellar astrocytes is dependent on ERK and p38 activity. Astrocytes were maintained for 20 min in the presence or absence of the MEK inhibitor U0126 (10 µM), the p38 inhibitor SB202190 (10 µM), or both prior to adding BzATP (300 µM, 30 min: **A**). In another set of experiments, astrocytes pretreated with or without U0126 were stimulated with EGF (100 ng/mL, 30 min: **B**). *Dusp1* and *Gapdh* expression was evaluated by qPCR as described in Fig. 3, normalized to that of *Gapdh*, and the values represent the means ± SEM from three independent experiments performed on different cultures: ****p < 0.0001; **p < 0.01; *p < 0.05

To assess the contribution of ERK1/2 to the *Dusp1* transcription triggered by the two agonists, we tested the effect of the mitogen extracellular activated kinase (MEK) inhibitor, U0126 (Fig. 4). Pretreatment (30 min) of cerebellar astrocytes with U0126 (10 µM) significantly dampened *Dusp1* expression, although a component resistant to MEK inhibition was observed after longer incubations with the nucleotide (Fig. 4A). Considering that P2X7R stimulation also activates p38 MAP kinase in astrocytes, we analyzed the sensitivity of BzATP-triggered *Dusp1* transcription to the p38 inhibitor, SB202190. Pretreatment of astrocytes with SB202190 (10 µM, 30 min) also reduced *Dusp1* expression, and again, an insensitive component was detected after 30 min and 1 h in the presence of BzATP. Preincubation of astrocytes with the two inhibitors fully blocked the BzATP-induced induction of *Dusp1* mRNA expression (Fig. 4A). Hence, the ERK1/2 and p38 MAP kinases appear to be involved in the induction of *Dusp1* transcription triggered by P2X7R stimulation of cerebellar astrocytes. However, the expression of *Dusp1* induced by EGF was exclusively dependent on ERK1/2 activation as it was prevented by inhibiting MEK (Fig. 4B).

### *Dusp1* expression induced by P2X7R-triggered ERK activation depends on EGFR transactivation

**Fig. 5:**
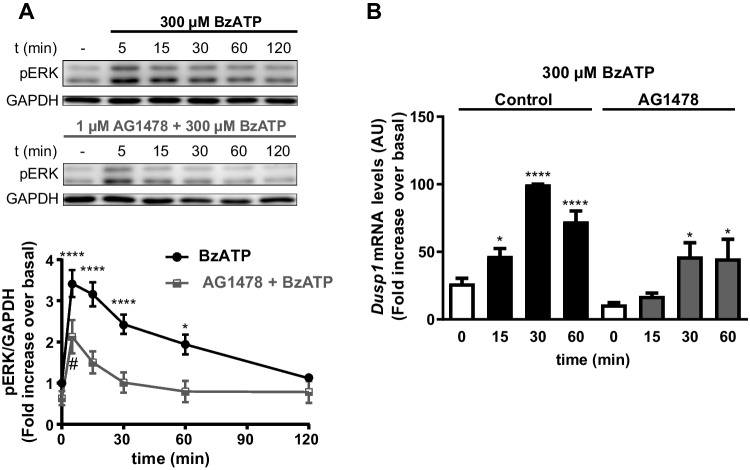
Inhibition of EGFR tyrosine kinase activity modifies ERK1/2 phosphorylation and the induction of *Dusp1* expression triggered by BzATP in rat cerebellar astrocytes. Astrocytes were maintained in the presence or absence of the EGFR inhibitor AG1478 (1 µM, 20 min) before being stimulated with the nucleotide for the times indicated, and the phosphorylated ERK1/2 in the cells was detected in immunoblots (**A**, representative immunoblots are shown). The graph shows the increase in ERK phosphorylation with respect to the basal levels detected in control conditions (unstimulated cells) and normalized to the corresponding GAPDH levels. Pretreatment of astrocytes with AG1478 also affected the induction of *Dusp1* transcription elicited by BzATP (**B**). Cells were maintained in the presence or absence of AG1478 (1 µM, 20 min) before stimulation with the nucleotide, and the *Dusp1* and *Gapdh* mRNA transcripts expressed were detected by qPCR. *Dusp1* expression was normalized to that of *Gapdh* and the values represent the mean ± SEM of the data obtained from at least three experiments performed on different cultures: ****p < 0.0001; #/* < 0.05

Considering the intense cross-talk between tyrosine kinase and nucleotide receptors established in the literature [42–44], we examined the possible contribution of EGFR to the ERK activation displayed by BzATP in cerebellar astrocytes. We analyzed the effect of the EGFR inhibitor, AG1478, on the ERK activation triggered by P2X7R stimulation (Fig. 5A), which altered the pattern of ERK activation produced by the nucleotide. In control conditions, maximal BzATP-induced ERK activation was reached after 5–15 min of stimulation, decaying slowly, and returning to basal levels 2 h after exposure to the nucleotide. However, EGFR inhibition made ERK activation more transient, peaking after a 5-min stimulation and reaching basal levels after a 30-min incubation with BzATP. These findings suggested that EGFR transactivation might be involved in regulating DUSP1 expression in these glial cells.

Inhibiting EGFR activity in astrocytes significantly reduced the *Dusp1* transcripts in these cells, but it did not prevent the increases elicited by BzATP stimulation (Fig. 5B). The AG1478 insensitive component could be attributed to p38 activation, although simultaneous inhibition of EGFR and p38 MAP kinase did not block *Dusp1* transcription. It is very interesting to note that prior exposure of astrocytes to the EGFR inhibitor completely prevented BzATP-induced Dusp6 expression (results not shown). Hence, EGFR transactivation appears to be essential for the activation of ERK produced by P2X7R stimulation, and for the ensuing induction of *Dusp1 and Dusp6* expression.

### P2X7 and BDNF receptor stimulation regulates DUSP1 levels in rat cerebellar granule neurons

**Fig. 6:**
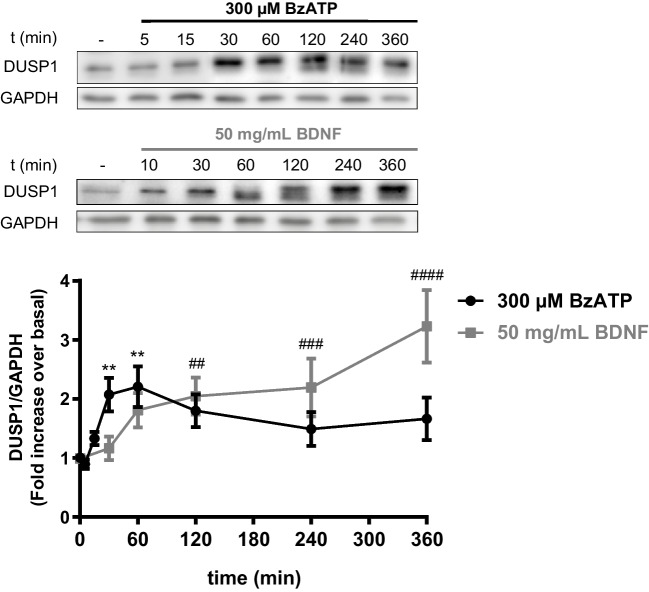
BzATP and BDNF increase the endogenous levels of DUSP1 protein in rat cerebellar granule neurons. Cerebellar neurons were stimulated with BzATP (300 µM) or BDNF (50 ng/mL) for the times indicated, and the DUSP1 and GAPDH in the total lysates of the cells was assessed in immunoblots for each effector. The graph shows a quantification of the time courses relative to those of the control cells (unstimulated) and normalized to the corresponding GAPDH levels. The data are represented as the means ± SEM from at least three independent experiments performed on different cultures: ####*p* < 0.0001; ###*p* < 0.001; ##/**p < 0.01

As BzATP and EGF stimulation triggered ERK activation and regulated the endogenous DUSP6 levels in cerebellar granule neurons [23], they might also modulate the levels of DUSP1 protein. Accordingly, granule neurons were stimulated with BzATP (300 µM), and the levels of DUSP1 phosphatase were assessed in Western blots. P2X7R stimulation increased the levels of DUSP1 but to a lesser extent than in astrocytes (Fig. 6), with maximal levels achieved after a 30-min stimulation and unlike astrocytes, this increase in DUSP1 persisted at all the time points analyzed. In granule neurons, EGFR stimulation did not affect the DUSP1 levels, yet BDNF stimulation (50 ng/mL) did provoke a time-dependent increase of this phosphatase, with a maximal 3-fold increase over the basal levels observed after a 6-h exposure to this neurotrophin (Fig. 6).

**Fig. 7:**
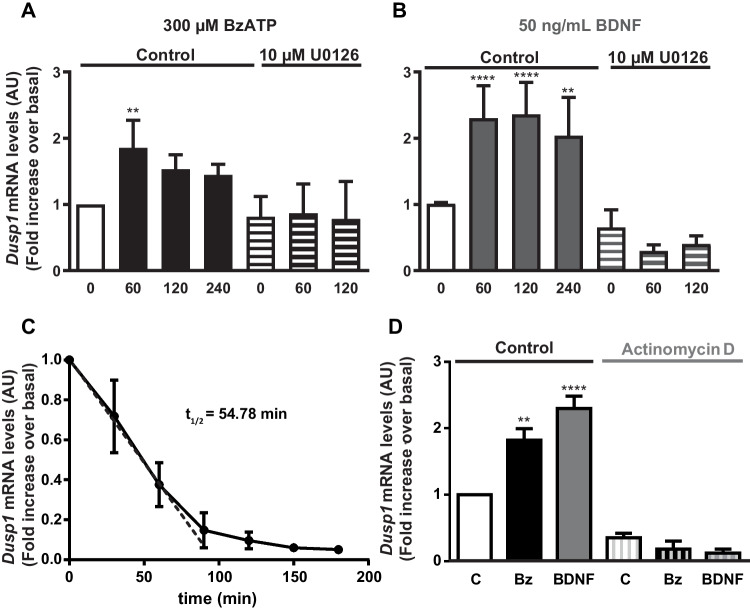
BzATP and BDNF stimulation increase the *Dusp1* mRNA transcript levels in rat cerebellar granule neurons. Cells were treated with the nucleotide (300 µM: **A**) or the neurotrophin (50 ng/mL: **B**) for the times indicated, and the *Dusp1* and *Gapdh* transcripts in the total mRNA isolated from the cells were quantified by RT-qPCR. Where indicated, cells were pre-incubated in the presence or absence of U0126 as described in Fig. 4. **C** *Dusp1* expression is regulated at the transcriptional level. Granule neurons were treated with actinomycin D (10 µM) for the times indicated, and the *Dusp1* mRNA transcripts were measured by qPCR and normalized to those of *Gapdh*. The half-life (*t*_1/2_) of the *Dusp1* mRNA transcripts was calculated by linear regression. **D** Cells were pretreated with actinomycin D (10 µM) for 30 min, stimulated for 1 h with BzATP (300 µM) or BDNF (50 ng/mL), and the *Dusp1* transcripts were quantified. *Dusp1* expression was normalized to that of *Gapdh* and the data are presented as the means ± SEM from at least three independent experiments performed on different cultures: ****p < 0.0001; **p < 0.01

The expression of *Dusp1* by granule neurons after exposure to BzATP or BDNF was assessed by RT-qPCR, demonstrating the increase in DUSP1 transcripts following stimulation with either agonist, which persisted for at least 4 h in their presence (Fig. 7 A and B). In both cases, the induction of *Dusp1* transcription was impeded by pretreatment with the MEK inhibitor, U0126, revealing *Dusp1* expression in granule neurons was dependent on ERK activation. The half-life of the *Dusp1* transcripts was determined in the presence of actinomycin D to block de novo transcription, and the loss of *Dusp1* mRNA was reflected in a half-life of 54.78 min (Fig. 7C). Neither BzATP nor BDNF stimulation altered the half-life of these transcripts, and pretreatment with actinomycin D completely blocked the increase in *Dusp1* expression produced by BzATP and BDNF (Fig. 7D).

### Post-translational regulation of DUSP1 in rat cerebellar astrocytes and granule neurons

**Fig. 8:**
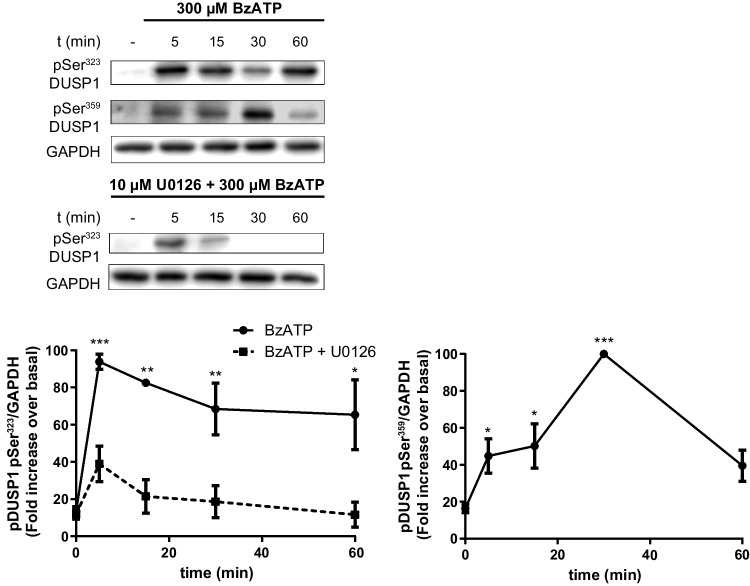
P2X7R stimulation induced Ser^323^ and Ser^359^ phosphorylation of DUSP1 in cerebellar astrocytes. Cells were exposed to BzATP (300 µM) for the times indicated, and the presence of phopho-DUSP1 and GAPDH was analyzed in immunoblots. In another set of experiments, cells were maintained in the presence or absence of U0126 (10 µM, 20 min) prior to nucleotide stimulation. The graphs show the quantification over time relative to the maximal phosphorylation (100%) in each experiment after normalization to the corresponding GAPDH levels. The data represent the means ± SEM of three independent experiments performed on different cultures: ***p < 0.001; **p < 0.01; *p < 0.05

We also studied other mechanisms that might be implicated in the regulation of DUSP1 activity in cerebellar cells, such as distinct post-translational modifications. The best-characterized are the phosphorylation events triggered by their own substrates, MAPKs. DUSP1 can be phosphorylated at different residues, driving its stabilization or degradation [45, 46]. To assess this hypothesis, we analyzed the presence of Ser^323^ and Ser^359^ phospho-DUSP1 in extracts of astrocytes stimulated with BzATP or of granule neurons treated with BDNF. Astrocyte stimulation with BzATP induced a rapid increase in Ser^323^ DUSP1 phosphorylation (Fig. 8), an event associated with the ubiquitin ligase recruitment that targets DUSP1 for proteasomal degradation. The phosphorylation of this residue was reduced significantly when the cells were pretreated with the MEK inhibitor, U0126. The remaining phosphorylation observed after short-time exposures (5–15 min) must be achieved through another protein kinase, like p38. Interestingly, despite the increases in Ser^323^ DUSP1 phosphorylation observed, the DUSP1 levels still remained high after a 1-h stimulation with the nucleotide (Fig. 2). Indeed, P2X7R stimulation also increased Ser^359^ DUSP1 phosphorylation in association with DUSP1 stabilization (Fig. 8). Astrocyte stimulation with EGF also enhanced the presence of the two phosphorylated isoforms of DUSP1 (results not shown).

**Fig. 9:**
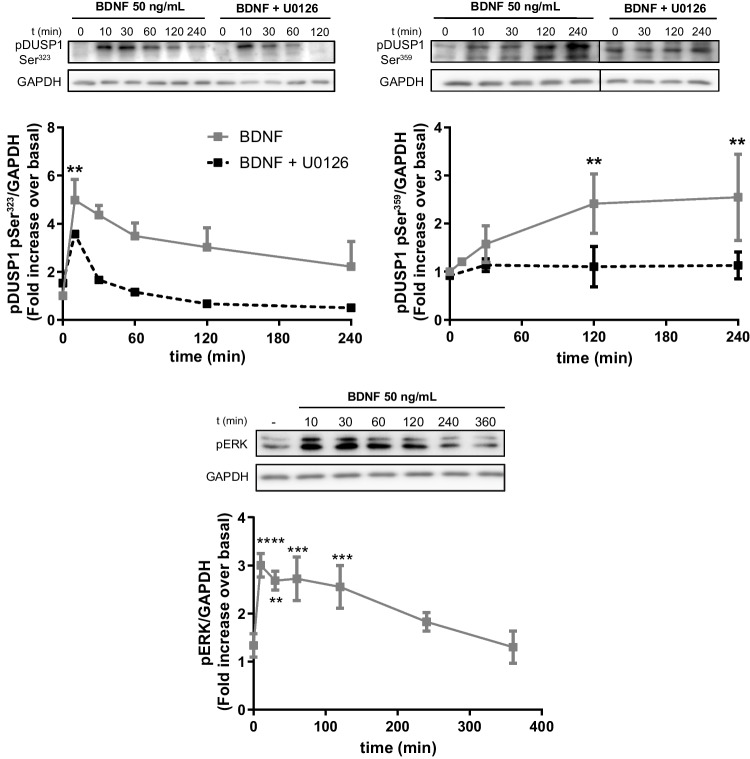
BDNF induces Ser^323^ and Ser^359^ phosphorylation of DUSP1 in cerebellar granule neurons. Cells were treated with BDNF (50 ng/mL) for the times indicated and the presence of phopho-DUSP1, phospho-ERKs, and GAPDH was assessed in immunoblots. In another set of experiments, granule neurons were maintained in the presence or absence U0126 (10 µM, 20 min) prior to neurotrophin stimulation. The graphs show the quantification over time obtained by normalizing to the corresponding GAPDH levels in which the data are presented as the means ± SEM from three independent experiments performed on different cultures: **p < 0.01

The pattern of DUSP1 phosphorylation provoked by stimulating granule neurons with BDNF was also assessed (Fig. 9). BDNF induced a rapid increase in Ser^323^ phosphorylation (after a 10-min stimulation), which decreased slowly following longer incubations. This neurotrophin also increased Ser^359^ DUSP1 phosphorylation in a time-dependent pattern resembling that observed for the total DUSP1 protein. Both phosphorylation events were dependent on ERK1/2 activation, which was maintained over basal levels in the presence of BDNF (Fig. 9). As expected, DUSP1 phosphorylations were dampened considerably by MEK inhibition, although a component of Ser^323^ DUSP1 phosphorylation insensitive to the MEK inhibitor was also detected, as seen in glial cells. To identify the kinases responsible for Ser^323^ DUSP1 phosphorylation after a 10-min exposure to BDNF, we performed similar experiments with the PI3K and PKC inhibitors, LY294002 and Gö6976, respectively. Neither of these inhibitors hindered this phosphorylation (results not shown), indicating that the protein kinase mediating the phosphorylation remains unknown. We were unable to detect any phosphorylated DUSP1 in granule neurons exposed to BzATP.

Overall, these results indicate that the levels of DUSP1 in the two models studied, rat cerebellar astrocytes and granule neurons, are regulated on at least at two different levels: at the transcriptional level and through the stabilization of the newly synthesized protein.

### Prolonged inhibition of the DUSP1/DUSP6 phosphatases by BCI affects the survival of rat cerebellar cells

**Fig. 10:**
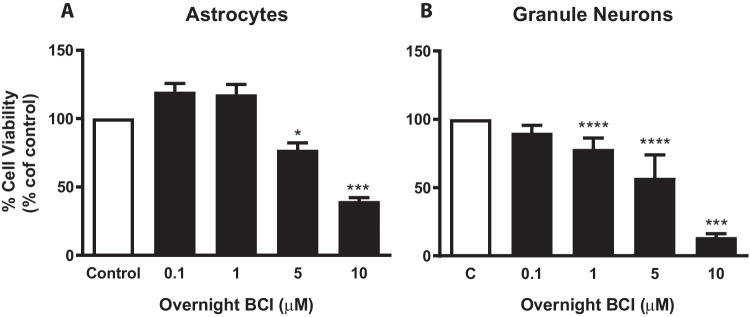
Long-term treatment of rat cerebellar cells with BCI is cytotoxic. Cytotoxicity was analyzed in MTT assays on astrocytes (**A**) and granule neurons (**B**) exposed to different concentrations of BCI for 24 h (see “Methods”). The values were normalized to those obtained from untreated cells (dose = 0, set as 100% viability) and the data represent the means ± SEM of three independent experiments performed on different cultures: ****p < 0.001; ***p < 0.001; *p < 0.05

To shed light on the physiological implications of DUSP activity in astrocytes and granule neurons, we tested the effect of prolonged DUSP1 and DUSP6 inhibition. The DUSP1/6 inhibitor BCI reduces cell viability in a dose- and time-dependent manner in a wide variety of tumor models, acting as an anti-tumorigenic agent [47–49], although it also exhibits pro-tumorigenic activity in other cellular models [50]. The potential toxicity of BCI was tested in cerebellar cells through MTT assays. Long exposure (24 h) of cerebellar cells to the DUSP inhibitor reduced cell viability in a concentration-dependent manner (Fig. 10). As expected, granule neurons were more sensitive than glial cells. These results corroborated that tight spatial–temporal regulation of DUSP activity is essential to adjust the MAP kinase activation required to maintain cell survival.

## Discussion

Purified cultures of cerebellar astrocytes and granule neurons have proved to be useful models to put together the elements of the intracellular signaling pathways activated by P2X7R. Prolonged P2X7R stimulation of cerebellar astrocytes and granule neurons does not affect cell survival but rather, sustained P2X7R activation with BzATP provokes neuroprotection and trophic effects in astrocytes under specific culture conditions [20, 21], potentially through the tight regulation of MAP kinases. Recent studies identified a protein phosphatase responsible for ERK1/2 inactivation in the cytosolic compartment as a new signaling target for P2X7R, the dual-specificity protein phosphatase DUSP6. The biphasic regulation of DUSP6 levels through P2X7R stimulation assured a fine-tuned time course of cytosolic ERK1/2 activation required for cell survival [23]. Here, we demonstrate that P2X7R stimulation also regulates the nuclear inducible protein phosphatase DUSP1 in cerebellar astrocytes and granule neurons. In cerebellar astrocytes, the expression of DUSP1 was controlled by P2X7 and EGF receptors, whereas EGFR had no such effect in granule neurons but rather the BDNF receptor-regulated DUSP1 expression along with P2X7R. The regulation of the DUSP1 phosphatase in astrocytes and neurons differs significantly as it was transient in astrocytes, yet it persisted in granule neurons, which anticipates different functions.

BzATP and EGF stimulation of astrocytes causes a rapid and transient increase in DUSP1 protein, reaching a maximum after a 1 h stimulation and then returning to basal levels 6 h later, as reported in macrophages and immune cells [51–53]. These increases in DUSP1 protein were due to an induction of *Dusp1* transcription by the two effectors, the transcripts maintaining a similar half-life to that described previously (15–40 min) [52, 54, 55]. As in immune cells, different MAPKs participate in the transcriptional induction of *Dusp1* depending on the nature of the stimulus. ERK1/2 and p38 activity is required by cerebellar astrocytes to induce *Dusp1* transcription in response to P2X7R stimulation. However, the induction of *Dusp1* expression by EGFRs depends exclusively on the activation of ERK1/2. Moreover, the DUSP1 expression triggered by P2X7R stimulation is stronger than that elicited by EGFR. These results contrast with the regulation of DUSP6 phosphatase, which is more pronounced after EGFR stimulation. Interestingly, transactivation of EGFR is required for *Dusp1* and *Dusp6* induction by P2X7R, which corroborates the key role that EGFR exerts in cerebellar astrocytes [53].

Cerebellar astrocytes and macrophages share some regulatory mechanisms of nucleotide receptor signaling that might participate in inflammatory processes and tissue remodeling [56, 57]. In cerebellar astrocytes, PGE_2_ prevents UTP sensitive-receptor signaling from acting on EP3 receptors, thereby impairing the intracellular calcium mobilization, ERK1/2 activation, Akt, and cell migration induced by UTP. PGE_2_-sensitive EP3 receptors require EGFR transactivation to dampen P2Y signaling and the effects of PGE_2_ also occur in a pro-inflammatory context, as witnessed in astrocytes stimulated with bacterial LPS. The data presented here add another layer of cross-talk between the nucleotide and EGF receptors. In this case, EGFR transactivation is required for DUSP1 expression dampening of P2X7R signaling to prevent any MAPK overactivation that might occur as P2X7R does not desensitize in the continued presence of the agonist.

Another interesting level of cross-talk between EGF and P2X7 receptors was reported in neuroblastoma cells, in which P2X7R promoted cell proliferation that when blocked, favored differentiation to a neuronal phenotype. Serum withdrawal triggers EGFR-dependent activation of the PI3-kinase/Akt pathway in neuroblastoma cells, resulting in the phosphorylation of the Sp1 transcription factor and the induction of P2X7R expression, which promotes cell proliferation in the absence of trophic support [44]. Recently, studies performed with the DUSP1/DUSP6 inhibitor BCI showed that p38 activation also upregulates P2X7R levels in neuroblastoma cells, indicating that the DUSP1 phosphatase may negatively modulate P2X7R expression by dampening the p38 pathway [50].

It is interesting to note that the regulation of DUSP1 expression in rat cerebellar granule neurons differed from that in astrocytes, being closely associated with BDNF, as described in murine cortical neurons [34]. The effect of BDNF on *Dusp1* expression was more prominent than that triggered by BzATP, although longer exposure to BzATP and BNDF increased DUSP1 levels at all the time periods tested. The increase in DUSP1 protein was in good agreement with the *Dusp1* transcripts detected. Studies performed with actinomycin D confirmed that the increase in *Dusp1* transcripts was due to the induction of gene transcription, and it was fully dependent on ERK1/2 activation. The half-life of *Dusp1* transcripts in granule cells was longer than in astrocytes, which seems to be related to granule neuron transcription since the half-life of *Dusp6* transcripts was also higher than that observed in astrocytes [23]. One possible explanation would be that higher levels of DUSP mRNA and protein are needed for neurons to fine regulate the MAP kinase activities elicited by the depolarizing potassium concentrations present in the culture medium to maintain neuronal survival. Accordingly, mRNA stabilization could take place in these neurons, perhaps driven by the presence of mRNA-stabilizing proteins (e.g., Human antigen R and Nuclear Factor 90) that bind to *Dusp1* transcripts and enhance its half-life [58]. Likewise, the contribution of different MAPK kinases to the induction of *Dusp1* transcription might also explain this apparent discrepancy, as only ERK activity but not p38 is required for *Dusp1* expression in granule neurons.

In addition to the primary role that MAP kinases play on *Dusp1* transcriptional induction, they also participate in the post-translational modifications of the protein, and this provides an additional mechanism to regulate this phosphatase [45]. The post-translational modification of the DUSP1 protein was also explored here, although we were unable to conclude these studies due to a lack of efficacy of new antibody batches recognizing both the unphosphorylated and phosphorylated forms of DUSP1. The results obtained clearly show that ERK1/2 activities regulate DUSP1 phosphorylation at two residues, Ser^323^ and Ser^359^, which are involved in the degradation and stabilization of DUSP1 protein, respectively, in cerebellar astrocytes and granule neurons. Nevertheless, an insensitive component of Ser^323^ phosphorylation status of DUSP1 to MEK inhibitor was detected. In astrocytes, the remaining phosphorylation might be accounted for by p38 as BzATP provoked a marked increase in p38 phosphorylation, much higher than that obtained for the ERKs (results not shown). The p38 kinase is one of the first intracellular signaling molecules activated by P2X7R stimulation in macrophages [64] and astrocytes obtained from different brain areas [5, 65]. Thus, p38-dependent signaling is intimately linked to the physiology of P2X7R, and it may contribute to the cross-regulation of different intracellular signaling pathways, even regulating the magnitude and duration of ERK signaling. In granule neurons, the insensitive component to MEK inhibition might likely be mediated by other kinases like PKCδ, as described in a hippocampal cell line and cortical neurons exposed to high concentrations of glutamate [40].

Ser^323^ DUSP1 phosphorylation is thought to drive recruitment of the ubiquitin ligase SCFSKP2 and hence, proteasomal degradation of this phosphatase [46, 66]. However, the increase in Ser^323^ phosphorylation was not related to the amount of protein detected. After a 1-h stimulation of astrocytes with BzATP, DUSP1 phosphorylation remained very high, and maximal protein levels were reached. Therefore, ubiquitin ligase recruitment may require additional factors, such as the simultaneous phosphorylation at Ser^296^ or other residues involving the opposite effects [66]. In fact, BzATP treatments also induced Ser^359^ DUSP1 phosphorylation that might contribute to the protein stabilization [45], albeit with a different time course that peaked at 30 min of stimulation. In cerebellar granule neurons, BDNF but not BzATP stimulation triggered the Ser^359^ DUSP1 phosphorylation albeit later, and it augmented at longer exposure times [40, 45]. In primary cultures of murine cortical neurons, the use of mutants of the different DUSP1 serine residues susceptible to phosphorylation by ERK confirmed that BDNF-mediated DUSP1 stabilization occurs through Ser^359^ phosphorylation [34]. Together, these results suggest that the regulation of DUSP1 levels by phosphorylation is complex and might be the result of several mechanisms acting simultaneously, dependent on the kinetics of MAPK activation or that of other kinases. Additional experiments will be required to identify other residues susceptible to phosphorylation that are also implicated in the regulation of DUSP1 in cerebellar astrocytes and granule neurons.

Although the physiological meaning of DUSP1 regulation in neural cells remains unclear, the balance between the mechanisms of activation/deactivation of MAPK signaling is essential for cell survival and neuronal differentiation, as prolonged inhibition of DUSP1 and DUSP6 activity by long-term exposure to BCI clearly compromises cell viability. Studies on primary murine cortical neuron cultures demonstrated that BDNF promoted dendritic arborization and axon branching by regulating DUSP1 levels and identified JNKs as their preferred substrates associated to cytoskeletal dynamics [34, 67].

In addition to the influence of DUSP phosphatases on neuronal differentiation, finely tuned regulation of MAP kinase signaling is also necessary to maintain neuronal survival and prevent death in the face of pro-apoptotic or stressful stimuli. DUSP1 expression is induced after hypoxia and ischemic events. The increase in DUSP1 activity impedes p38 and JNKs hyperphosphorylation and the activation of the pro-apoptotic program that leads to neuronal death and inflammatory responses [59–62]. In sympathetic neurons, the withdrawal of trophic factors like NGF induces *Dusp1* expression in a JNK-dependent manner, which participates in a negative feedback loop to neutralize JNK activation and promote neuronal survival [63]. In granule neurons and cerebellar astrocytes, DUSP1 activity could also prevent the overactivation of ERK and p38 elicited by pro-apoptotic stimuli, like UV irradiation or an oxidative environment. In this overactivation of MAP kinases is closely associated with the dysregulation and loss of DUSP activity, either due to their dampened expression or catalytic inactivation, as occurs under excitotoxic conditions or following exposure to oxidative/genotoxic stress [40, 68–70]. We previously reported the P2Y_13_ nucleotide receptor elicits neuroprotection in the face of genotoxic stress in cerebellar granule neurons. P2Y_13_ activation induces ERK-dependent expression of *Dusp2*, which in turn counteracts the nuclear accumulation of phosphorylated p38 [71]. The present findings point to DUSP1/DUSP6 inhibition as the cause of BCI-induced toxicity in both cerebellar astrocytes and granule neurons. Therefore, restoring DUSP levels might prevent the apoptotic consequences of MAP kinase overactivation [71].

The possible neuroprotective role of DUSPs is also manifested in neurodegeneration, following brain damage and in neurological disorders, in which drastic changes of the activity and expression of DUSPs have been described [72–76].

**Fig. 11:**
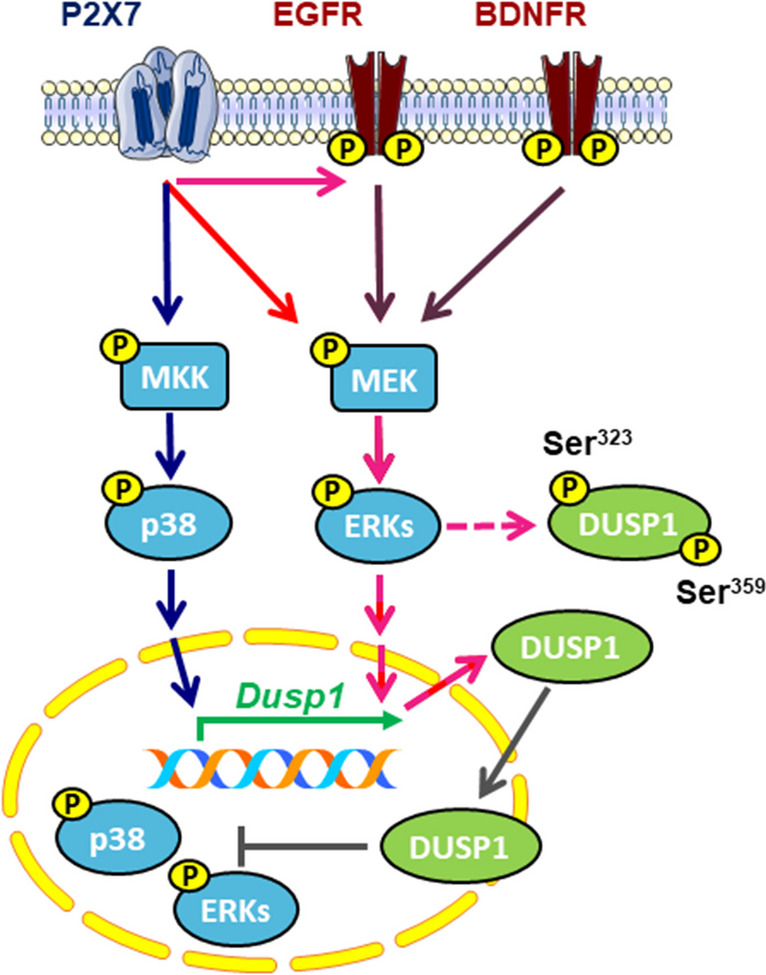
Scheme summarizing the regulation of DUSP1 expression by P2X7R and the tyrosine kinase receptors, EGFR and BDNFR, in rat cerebellar cells. Dusp1 induction is dependent on ERK1/2 and p38 activation in astrocytes. In these glial cells, EGFR is required for ERK activation and Dusp1 induction displayed by P2X7R. By contrast, DUSP1 expression is exclusively dependent of ERK activity in cerebellar granule neurons. DUSP1 protein levels might be also controlled by phosphorylation processes at different residues

To conclude, our findings show that nucleotide receptors, specifically the P2X7R, and the EGF and BDNF Trk receptors, regulate the endogenous levels of the DUSP1 protein phosphatase in rat cerebellar cells, astrocytes, and granule neurons (Fig. 11). DUSP1 represents an additional convergence point for nucleotides and growth factors/neurotrophins involved in MAPK signaling. Not only do they coincide in the activation of MAPK signaling but also, in its deactivation. Although this regulation might take place under multiple circumstances, the preferential location of astrocytes in certain brain areas will make it essential to define the levels of expression of these receptors. Moreover, the cellular context in which the glial cells and neurons interact in such regions is likely to modify these interactions.

## Data Availability

The data presented in this study is available from the corresponding author upon reasonable request.
